# Evaluating the Impact of Handling and Logger Attachment on Foraging Parameters and Physiology in Southern Rockhopper Penguins

**DOI:** 10.1371/journal.pone.0050429

**Published:** 2012-11-21

**Authors:** Katrin Ludynia, Nina Dehnhard, Maud Poisbleau, Laurent Demongin, Juan F. Masello, Petra Quillfeldt

**Affiliations:** 1 Max-Planck Institute for Ornithology, Vogelwarte Radolfzell, Radolfzell, Germany; 2 Animal Demography Unit, University of Cape Town, Cape Town, South Africa; 3 Department Biology – Ethology, University of Antwerp, Antwerp, Belgium; University of Roehampton, United Kingdom

## Abstract

Logger technology has revolutionised our knowledge of the behaviour and physiology of free-living animals but handling and logger attachments may have negative effects on the behaviour of the animals and their welfare. We studied southern rockhopper penguin (*Eudyptes chrysocome*) females during the guard stage in three consecutive breeding seasons (2008/09−2010/11) to evaluate the effects of handling and logger attachment on foraging trip duration, dive behaviour and physiological parameters. Smaller dive loggers (TDRs) were used in 2010/11 for comparison to larger GPS data loggers used in all three seasons and we included two categories of control birds: handled controls and PIT control birds that were previously marked with passive integrative transponders (PITs), but which had not been handled during this study. Increased foraging trip duration was only observed in GPS birds during 2010/11, the breeding season in which we also found GPS birds foraging further away from the colony and travelling longer distances. Compared to previous breeding seasons, 2010/11 may have been a period with less favourable environmental conditions, which would enhance the impact of logger attachments. A comparison between GPS and TDR birds showed a significant difference in dive depth frequencies with birds carrying larger GPS data loggers diving shallower. Mean and maximum dive depths were similar between GPS and TDR birds. We measured little impact of logger attachments on physiological parameters (corticosterone, protein, triglyceride levels and leucocyte counts). Overall, handling and short-term logger attachments (1–3 days) showed limited impact on the behaviour and physiology of the birds but care must be taken with the size of data loggers on diving seabirds. Increased drag may alter their diving behaviour substantially, thus constraining them in their ability to catch prey. Results obtained in this study indicate that data recorded may also not represent their normal dive behaviour.

## Introduction

Logger technology has revolutionised our knowledge on the behaviour and physiology of free-living animals (see [1], [2] for review), especially on seabirds that are usually difficult to study away from their colonies (see [3]). However, possible negative effects on the behaviour, physiology or survival of the birds have to be considered in any study where animals are disturbed, handled or equipped with tracking devices [2], [4]–[7]. Besides the ethical considerations regarding the animals’ welfare and their survival, changes in the animals’ behaviour induced by research activities will cause the research results to be biased and not represent the normal behaviour which the study focussed on [6], [8], [9]. A meta-analysis by Barron et al. [7] showed that many studies on birds find negative device effects. For seabirds, the number of publications dealing with potential impacts of these devices on the behaviour and physiology of study animals are rather limited albeit their extensive usage [10]. One reason for that is often the lack of comparable data from unequipped control animals [10], [11]. A way of assessing the impact of logger deployment is the comparison between different device sizes and weights [12], [13] as well as the comparison between internally and externally attached devices [11], [14].

Possible impacts of handling and logger attachments may include changes in foraging and diving behaviour, changes in time budgets as well as in physiological conditions, for example hormone levels or energy expenditure. Attachments can also alter breeding success and reduce survival rates (see [10]). These factors can also be linked and one might be the proximate cause of another. For example, a higher work load can lead to longer foraging trips, thus higher energy expenditure, which can result in reduced mass gain and muscular damage [15]. However, birds can also compensate for the higher energy expenditure, for example by changing their diving behaviour [16].

Most of the early studies using logger technologies were conducted on penguins and more than half of the publications defined by Vandenabeele et al. [10] as directly assessing logger impacts deal with this group of birds. These studies have mostly focused on changes in foraging and diving behaviour. More recently, technologies allow measuring physiological parameters during foraging (see [17] for review), and several studies have tried to assess physiological changes that occur due to logger attachments (e.g. [15], [18], [19]).

Physiological changes may be caused by handling stress and the attachment of a device which can affect baseline corticosterone levels and leucocyte counts such as the ratio between heterophils (or equivalently used granulocytes) and lymphocytes and the number of leucocytes per 10,000 red blood cells ( = RBC) (e.g. [20], [21]). Additionally, higher foraging costs due to the attachment of a device could affect plasma triglycerides that are indicative of the nutritional state (e.g. [22], [23]), and plasma protein levels, which increase with physical exercise ([24] and literature therein). Thus, handling and carrying a device could add stress to the bird, potentially increase foraging costs and decrease foraging success. We here define stress as any disturbance of homeostasis, which is accompanied by physiological changes that aim to re-establish homeostasis [25]. Long-term stress can lead to immune suppression and can even be fatal [26], [27]. Here, we tested whether handling and the attachment of different size data loggers have a negative impact on breeding southern rockhopper penguins (*Eudyptes chrysocome*). We considered potential effects on the foraging behaviour and diving behaviour as well as on physiological conditions (corticosterone, protein, triglyceride levels and leucocyte counts). Using data from three consecutive breeding seasons, we further tested for differences between breeding seasons as environmental conditions may influence the level of logger effects [11], [28].

If bird handling and logger attachment had an effect in our study, we predicted to see 1) longer foraging trip duration in logger-equipped birds compared to control birds and 2) different diving behaviour in relation to logger size with shallower dive depth but more frequent dives in birds with larger devices. We further predict that 3) handled controls re-establish homeostasis and recover to pre-capture stress levels over one foraging trip, while GPS birds retain higher stress levels. We therefore expected logger-equipped birds to show an increase in baseline corticosterone levels after foraging, indicating higher foraging costs [29], [30], and an increase in granulocyte/lymphocyte ratios (G/L ratio) as well as a decrease in leucocytes per 10,000 RBC over a foraging trip [20], [31]. Furthermore, we predicted 4), lower triglyceride and higher protein levels in logger-equipped birds compared to control birds after foraging, indicating decreased foraging success and increased physical exercise.

## Methods

### Ethics Statement

The study was approved by the Environmental Planning Department of the Falkland Islands Government (Research Licenses No: R17/2007, R12/2008, R05/2009). Potential impact of logger attachment is evaluated in the following analyses. Handling time was kept to a minimum, birds were taken out of sight of other breeding birds to keep disturbance in the colony low and the bird’s eyes were covered (see [6]).

### Study Site

The study took place at the New Island Nature Reserve (51°43′S, 61°17′W), one of the most westerly located islands of the Falkland Islands/Islas Malvinas in the Southwest Atlantic. More than 5,000 breeding pairs of southern rockhopper penguins breed at New Island [32]. The study was carried out in three consecutive breeding seasons (2008/09 to 2010/11) during the guard stage of southern rockhopper penguins in December (thus referred to as 2008, 2009 and 2010). During this stage, female birds leave the colony for foraging and chick provisioning [33]–[35], while males guard the chicks.

### Logger Types

In all three breeding seasons, GPS-temperature-depth data loggers (GPS-TD, earth&Ocean technologies, Kiel, Germany) were used, recording the GPS positions, dive depth (using a pressure sensor) and temperature (see [35], [36] for further details). Dimensions of the GPS data loggers were 96 mm length, 39 mm width and 26.5 mm height, thus having a cross-sectional area of 10.3 cm^2^ which represents about 6–10% of the birds’ cross-section (breast circumference of 105 females during Dec and Jan 2006−2009: mean 40.1 cm, range 36.0−47.0 cm). Devices weighed 75 g, thus being less than 3% of the body mass of females during guard (mean 2642 g, range 2120−3170 g; this study). The GPS data loggers were enclosed in a waterproof, hydrodynamically shaped housing and were attached to the lower back of the penguins using Tesa® tape, following Wilson et al. [37]. Loctite® was applied to cover the tape and prevent loosening of the tape ends.

In 2010, miniature dive loggers (DST micro TD, Star Oddi, Reykjavik, Iceland) were used on additional females during guard to study their diving behaviour. Dive loggers (hereafter referred to as TDRs) were 25.4 mm long and had a diameter of 8.3 mm, thus having a cross-sectional area of 0.54 cm^2^, between 0.3 and 0.5% of the birds’ cross-section. TDRs weighed 3.3 g, 0.1% of the females’ body mass. TDRs were attached to the lower back using Tesa® tape and Patex® rubber glue that was peeled off after recovering the device.

### Logger Attachment

Female rockhopper penguins were caught using a long pole with a metal hook to grab the birds around their legs and by hand while birds were standing next to their partners at the nest site. Sexes were distinguished by the size of the bill [38]. After catching the bird, its head was covered and the animal was held in a tight position by one person while the other person took a blood sample and morphometric measurements (bill sizes, flipper length and mass). A passive integrative transponder (PIT; 23 mm length, Texas Instruments, USA) was inserted subcutaneously in the back of the bird between the scapulae. GPS data loggers or TDRs were attached and birds were returned to their nest sites. The deployment process, including blood sampling, tag insertion and data logger attachment, lasted between 10 and 20 minutes. Nests were checked daily to determine departure and return of the birds. An automated gateway system was installed during all breeding seasons. All birds from the study colony passed this system and for birds tagged with PITs, departure and arrival times and dates as well as body mass were recorded. Loggers were taken off when the female birds returned to their nest sites after one day at sea (in 2009 and 2010) and after three days (including 2−3 foraging trips) in 2008. Blood samples were taken in 2009 and 2010 after the return of the birds.

In 2008, ten birds were equipped with GPS data loggers of which one device was lost (logger fell off) and one device did not yield GPS information (see [35]). In 2009, eleven birds were equipped with GPS data loggers of which ten data loggers yielded GPS data and in 2010, 15 GPS data loggers were deployed and 13 contained useful data. Sample sizes used for individual analyses are given in the tables, as not all data sets were complete for all trips. In 2010, an additional 15 birds were deployed with TDRs.

In 2009 and 2010, ten control birds (hereafter referred to as handled control birds) were caught and handled the same way as logger-equipped birds except for the deployment of data loggers. Handled control birds allowed us to separate the impact of handling versus logger attachment. To control for handling effects, gateway readings on departure and return times were used to calculate trip durations of twenty randomly chosen birds in each breeding season. These random birds (referred to as PIT control birds) had been tagged with PITs in previous seasons and were not handled during the time of this study. Nevertheless, they cannot, strictly speaking, be considered non-handled as previous handling and PIT deployment could have long-term effects influencing their current behaviour [8], [39]. PITs have been used in many bird species and have not been found to have negative effects on survival or breeding success [9], [40]–[42], not even in small passerines [43]. We are therefore confident that these birds constitute a reliable control category in our study.

### Trip Duration

Departure and arrival times of all birds were determined by passage through the gateway system which read the PITs of all birds included in this study. This method allowed for a better comparison of logger-equipped birds with handled and PIT control birds, as for these last two categories, trip duration could only be determined by readings from the gateway system. Only in case of missing gateway readings, GPS positions and depth/temperature data were used to determine time at sea for GPS and TDR birds. Trip durations were compared between breeding seasons and between bird categories (GPS, TDRs, handled control and PIT control birds) using linear models (LM) and linear-mixed effect models fit by REML (LME) run in R 2.13.1 (package „nlme”, [44], [45]). Individual birds were used as random factors where several trips of the same bird were considered. Given are modelled means ± standard error (SE) and minimum and maximum trip durations.

### Foraging Parameters - GPS Birds

GPS data loggers were set to record GPS positions every two minutes and upon each surfacing after dives. Maximum distance to the colony was calculated for the furthest position away from the colony and distance travelled was calculated as the sum of all distances between positions. We calculated the underwater time (sum of all dives) as percentage of the trip duration (see [16], [46]).

### Dive Analysis GPS Data Loggers vs. TDRs

Depth recordings were taken every second in GPS data loggers and every three seconds in TDRs. All pressure data were analysed using a custom-written Matlab script and dive parameters were calculated following Mattern et al. [47]. For comparison between data logger types (GPS data loggers vs. TDRs in 2010), pressure data from GPS data loggers in 2010 were sub-sampled to three seconds. Dive parameters were compared between the two data logger types using LME in R, with bird identity and trip number as random factors. As dive duration was positively correlated with dive depth, depth was additionally used as a random factor when testing for differences in dive duration. Individual dives might not have been identified as such using a 3 s interval setting and longer dive durations (see results) might therefore represent a combination of several dives (see [48]). Post-dive periods were only considered when not greater than 180 seconds (see [49]) and used to calculate the dive efficiency (bottom time/(dive duration+post-dive period); following [50]). Dive depth distributions were compared using Chi-square test with dives in 10 m intervals (first interval 3–10 m).

### Corticosterone, Protein, Triglyceride Levels and Leucocyte Counts

Blood samples in 2009 and 2010 were taken from the brachial vein within 3 minutes after capture of the bird, using a 23-gauge needle and a heparinised syringe. Blood samples were stored on ice while still in the colony, and were centrifuged thereafter. Plasma was kept frozen until the analyses took place. Blood smears were prepared in the field with blood that was taken directly from the skin puncture with a capillary. Following Ruiz et al. [51], one drop of blood was smeared on a glass slide and air-dried. Samples were later fixed with methanol (100%) and stained with Giemsa prior to counting.

Corticosterone concentrations (in ng/ml) were determined from blood plasma at the IZW Berlin. Plasma volumes ranged from 15–50 µl and were extracted twice with 2 ml of tert-butyl methyl ether:petrolether (30∶70, v/v) for 30 min. After freezing at –80°C for 20 min, the organic phase was decanted, dried and resolved with 0.1 ml of 40% methanol. Corticosterone was quantified by using a micro-titre plate enzyme immunoassay. The extraction of different plasma volumes gave displacement curves that were parallel to the corresponding corticosterone standard curve. The calibration curves for the assay ranged from 0.2 to 100 pg/aliquot. The sensitivity of the assay was defined as two standard deviations from the signal given by the zero blank and was 0.5 pg/aliquot. The intra-assay coefficients of variation of four biological samples were 5.3, 5.6, 7.3 and 12.7% (n = 4 each). The inter-assay coefficients of variation of two biological samples were 14.9 and 20.2% (n = 8 and n = 7, respectively).

Plasma protein and triglyceride levels were determined using standard spectrophotometric test combinations (see [52]). We used 6 µl of plasma per determination, using the total protein reagent (n° 981826, Thermo Fisher Scientific) and protein standard sCal (n° 981831, Thermo Fisher Scientific) for calibration. For triglycerides, the procedure was repeated with the Triglycerides reagent (n° 981786, Thermo Fisher Scientific) and sCal. Absorptions were measured with a spectrometer at 540 nm wavelength each.

Blood smears (only data from 2010) were scanned with a light microscope (1000×, oil immersion) in a monolayer of blood cells. Differential leucocyte counts were accomplished along the short-axis of the slide to control for differences in the thickness of blood cells (see e.g. [53], [54]). A minimum of 100 leucocytes were counted per slide and distinguished as granulocytes (heterophils, eosinophils and basophils pooled together, see [54]), lymphocytes and monocytes, following Hawkey and Dennet [55]. We calculated the G/L ratio as the ratio between granulocytes and lymphocytes, which can be used alternatively to the H/L ratio as a measurement of stress (e.g. [54], [56]). Leucocyte numbers per 10,000 RBC were calculated by counting the number of all RBC in three microscopic visual fields and multiplying the average value (i.e. average number of RBC per microscopic field) with the number of the microscopic visual fields that were scanned until reaching 100 leucocytes.

Wilcoxon and Kruskal-Wallis Signed-Rank tests (run in R) were used to test for differences in physiological parameters before and after foraging between different bird categories (GPS, TDR and handled control birds). Possible changes within bird categories before and after foraging were tested using paired Wilcoxon Signed-Rank tests.

## Results

### Tip duration: Breeding Season and Device Differences

**Figure 1:**
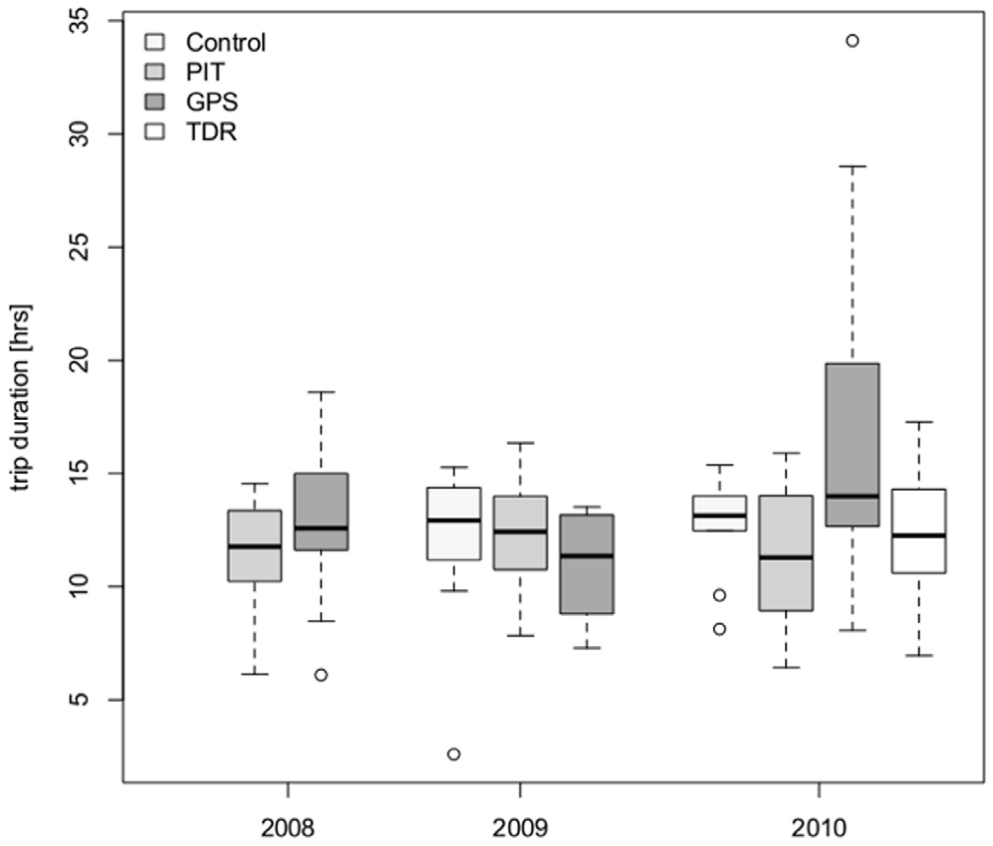
Trip duration of female southern rockhopper penguins from New Island. Comparison of trip duration (in hours) in three consecutive breeding seasons between different handling and logger attachment procedures. Given are median values (line), first and third quartiles (box) and the whiskers show 1.5 times the interquartile range. Outliers are plotted as dots.

No differences in trip durations were found in PIT control birds among the three breeding seasons (F_2,57_ = 0.961, p = 0.389; Table 1) nor in handled controls between 2009 and 2010 (F_1,18_ = 0.283, p = 0.601; Table 1). GPS birds in 2010 presented longer foraging trips than in the other two seasons, including four birds with overnight trips (Table 1). Trip durations in GPS birds were not significantly different between breeding seasons (t_31_ = 1.906, p = 0.066). However, testing the interaction of bird category and breeding season showed a significant difference in trip duration for GPS birds in 2010 when comparing GPS and PIT control birds in all breeding seasons (t_86_ = 2.343, p = 0.021) as well as when comparing GPS, handled controls and PIT control birds in 2009 and 2010 (t_80_ = 2.153, p = 0.034). In 2010, foraging trip durations of TDR birds were comparable to PIT and handled control birds (TDR; t_53_ = −0.186, p = 0.853; PIT: t_53_ = −0.837, p = 0.407 compared to handled controls; Table 1), only GPS birds had significantly longer foraging trips than all other bird categories (t_53_ = 2.289, p = 0.026; Table 1, Fig. 1).

**Table 1:** Trip duration (in hours) for female southern rockhopper penguins during guard stage from New Island, in three consecutive breeding seasons and averaged over the three breeding seasons (modelled means ± SE, range, n = number of birds (trips)).

	2008	2009	2010	average
PIT control	11.3±0.6	12.3±0.8	11.2±0.8	11.68±1.0
	6.13–14.55	7.83–16.40	6.42–15.90	
	n = 20	n = 20	n = 20	
Handled	NA	12.0±1.0	12.7±1.4	12.35±0.9
control		2.60–15.27	8.13–15.37	
		n = 10	n = 10	
GPS	12.9±1.6	10.9±2.3	17.0±2.1	14.02±1.1
	6.10–18.58	7.28–13.53	8.07–34.13	
	n = 9 (26)	n = 10 (10)	n = 15 (16)	
TDR	NA	NA	12.4±0.8	12.35±1.4
			6.95–17.28	
			n = 12 (12)	

PIT control: non-handled birds deployed with subcutaneous PITs in previous breeding seasons.

Handled control: birds handled but not equipped with any type of device.

GPS: birds equipped with GPS data loggers.

TDR: birds equipped with miniature TDR dive loggers.

### Foraging and Dive Parameters: Breeding Season Differences in GPS Birds

**Figure 2:**
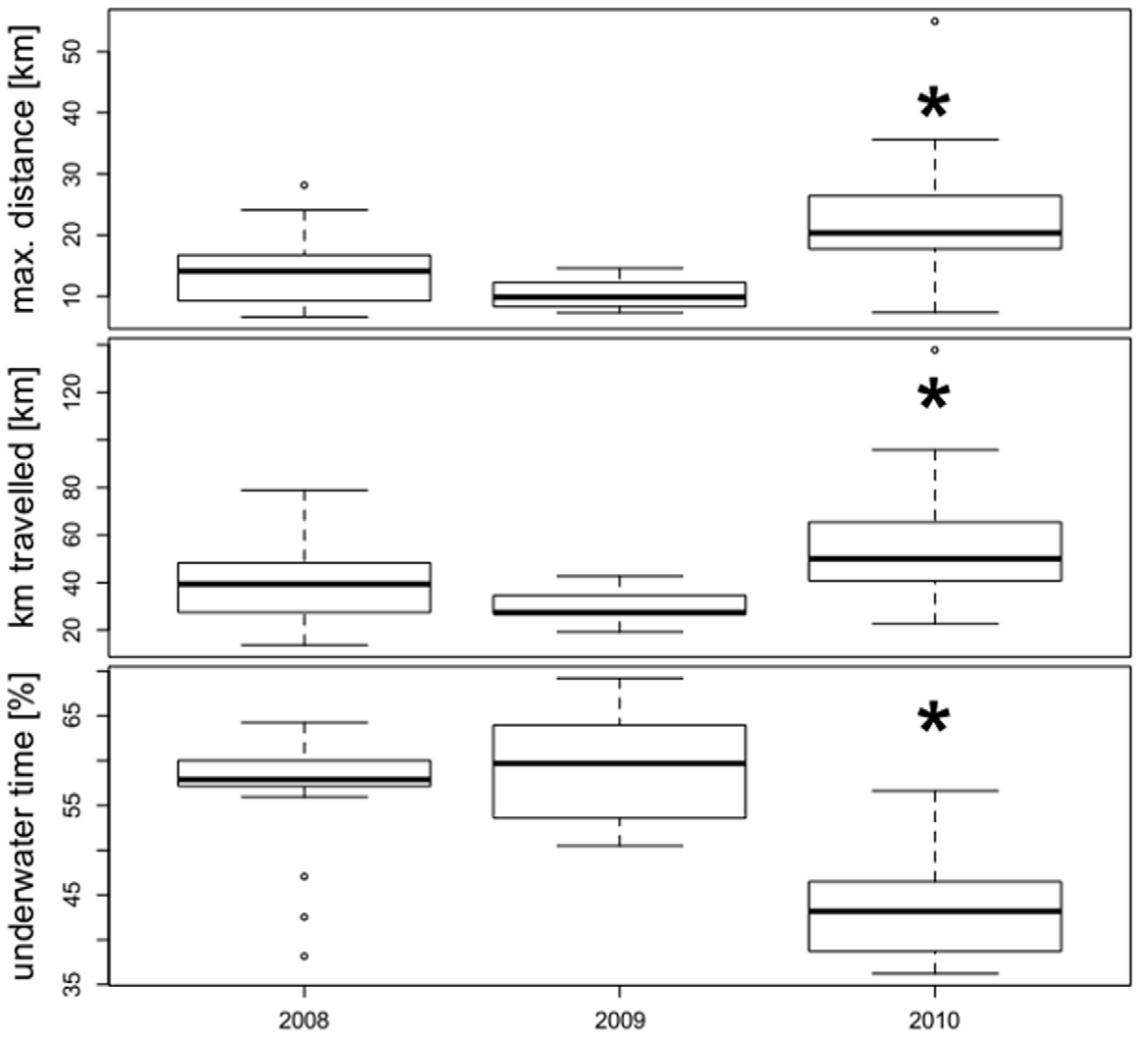
Foraging parameters of southern rockhopper penguins equipped with GPS data loggers. Maximum distance to the colony, distance travelled and underwater time (as % of trip duration) of GPS data logger-equipped females from New Island, in three consecutive breeding seasons. Stars mark significant differences for all three parameters in 2010 compared to previous breeding seasons (see Table 2).

Maximum distance from the colony and distance travelled were significantly greater for GPS birds in 2010 whereas underwater time was significantly lower in 2010 than in the two previous breeding seasons (Table 2 and Fig. 2). Dive parameters of GPS birds also showed some differences between breeding seasons with significantly greater maximum dive depth in 2010 (mean max. dive depth: 2008∶51.4±1.9; 2009∶58.0±3.6; 2010∶62.5±3.2 m; t_28_ = 3.439, p = 0.002) and longer mean dive durations in 2009 (2008∶66.4±2.3 s; 2009∶74.2±3.6 s; 2010∶68.4±3.4 s; t_28_ = 2.190, p = 0.04).

**Table 2:** Foraging parameters of female rockhopper penguins equipped at New Island with GPS data loggers (modelled means ± SE, range, n = number of birds (trips), significant results in bold).

	2008	2009	2010	2008 vs. 2010	2009 vs. 2010	2008 vs. 2009
Max distance	14.3±2.4	10.2±3.6	22.8±3.3	**t _28_ = 2.574, p = 0.016**	**t _28_ = 3.672, p = **	t _28_ = −1.165, p =
(km)	6.7–28.2	7.4–14.5	7.4–55.0		**0.001**	0.254
	n = 8 (22)	n = 10 (10)	n = 13 (14)			
Distance	39.3±4.8	29.9±8.2	59.6±7.4	**t _28_ = 2.738, p = 0.011**	**t _28_ = 3.408, p = **	t _28_ = −1.144, p =
travelled	13.7–78.9	19.2–42.8	22.7–137.8		**0.002**	0.262
(km)	n = 8 (19)	n = 10 (10)	n = 13 (14)			
Underwater	56.7±1.4	59.2±2.4	43.4±2.3	**t _27_ = 5.829, p<0.001**	**t _27_ = **−**5.843,**	t _27_ = 1.022, p =
time (% of	38.2–64.2	50.5–69.2	36.2–56.6		**p<0.001**	0.316
foraging trip)	n = 8 (21)	n = 10 (10)	n = 12 (12)			

### Dive Parameters - Comparison between GPS Data Loggers and TDRs

**Figure 3:**
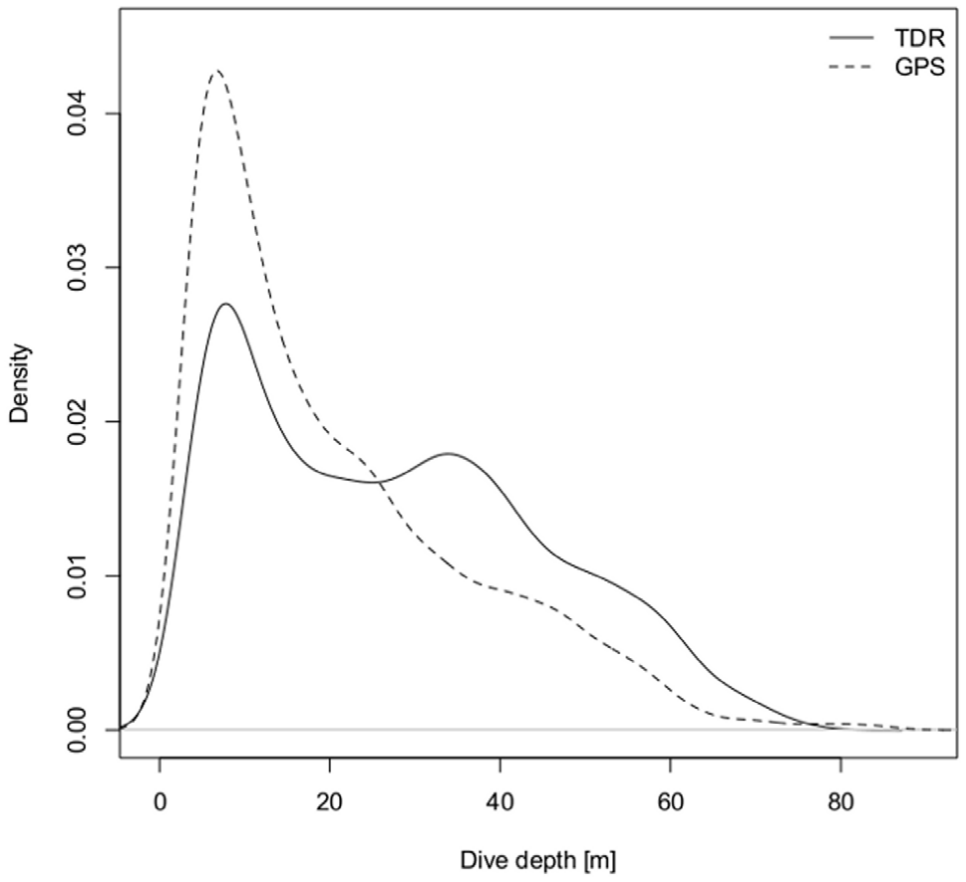
Dive depth distribution of southern rockhopper penguins. Density plot of dive depth distribution for females from New Island, carrying GPS data loggers and miniature TDR dive loggers in 2010.

The dive depth distributions were significantly different between GPS and TDR birds (Chi-square 423.3, df = 8, p<0.001). The density distribution showed that TDR birds dived more often to deeper depths whereas more than 50% of all dives by GPS birds were within the top 20 m (Fig. 3). Nevertheless, dive depth and maximum dive depth (per bird) were not significantly different between logger types (Table 3). The deepest dives recorded were conducted by GPS birds. TDR birds dived significantly longer (Table 3) but dive durations have to be handled with care. Sub-sampling the original 1 s dive data of GPS birds to 3 s intervals led to an increase in maximum dive durations (1 s intervals: 119.0±2.9 s vs. 3 s intervals: 126.3±9.8 s) and to a decrease in numbers of dives (1 s: 5246 dives vs. 3 s: 5151 dives). GPS birds presented more dives per trip but spent less time under water in relation to the trip duration (Table 3), due to some overnight trips with reduced diving activity during the night. Dive efficiency was significantly lower in GPS birds even though post-dive intervals were significantly longer in TDR birds (Table 3).

**Table 3:** Dive parameters of female rockhopper penguins equipped at New Island in 2010 with GPS data loggers and miniature TDR dive loggers (modelled means ± SE, significant results in bold).

	GPS (3 sec)	TDR	GPS vs. TDR
Total number of dives	5151	3751	
Mean dive depth (m)	23.1±2.4	29.7±3.4	t_22_ = 1.956, p = 0.06
Max dive (m)	64.3±2.4	68.6±3.4	t_22_ = 1.256, p = 0.222
Deepest dive (m)	86.5	78.0	
Mean dive duration (s)	**69.4±2.5**	**77.2±3.5**	**t_22_ = 2.215, p = 0.037**
Max duration (s)	126.3±9.8*	138.0±13.8*	t_22_ = 0.852, p = 0.404
Longest dive (s)	207*	249*	
Post-dive interval (s)	**28.66±3.12**	**36.79±4.42**	**t_22_ = 1.839, p = 0.079**
Dive-efficiency	**0.210±0.019**	**0.275±0.027**	**t_22_ = 2.424, p = 0.024**
Number of dives per trip	396.4±52.6	312.6±74.4	t_22_ = 1.126, p = 0.272
Underwater time (% of foraging trip)	42.7±3.6	50.1±5.2	t_23_ = 0.423, p = 0.168

* max dive durations are overestimated due to the 3 s interval in pressure data (see results).

### Corticosterone, Protein, Triglyceride Levels and Leucocyte Counts

**Figure 4:**
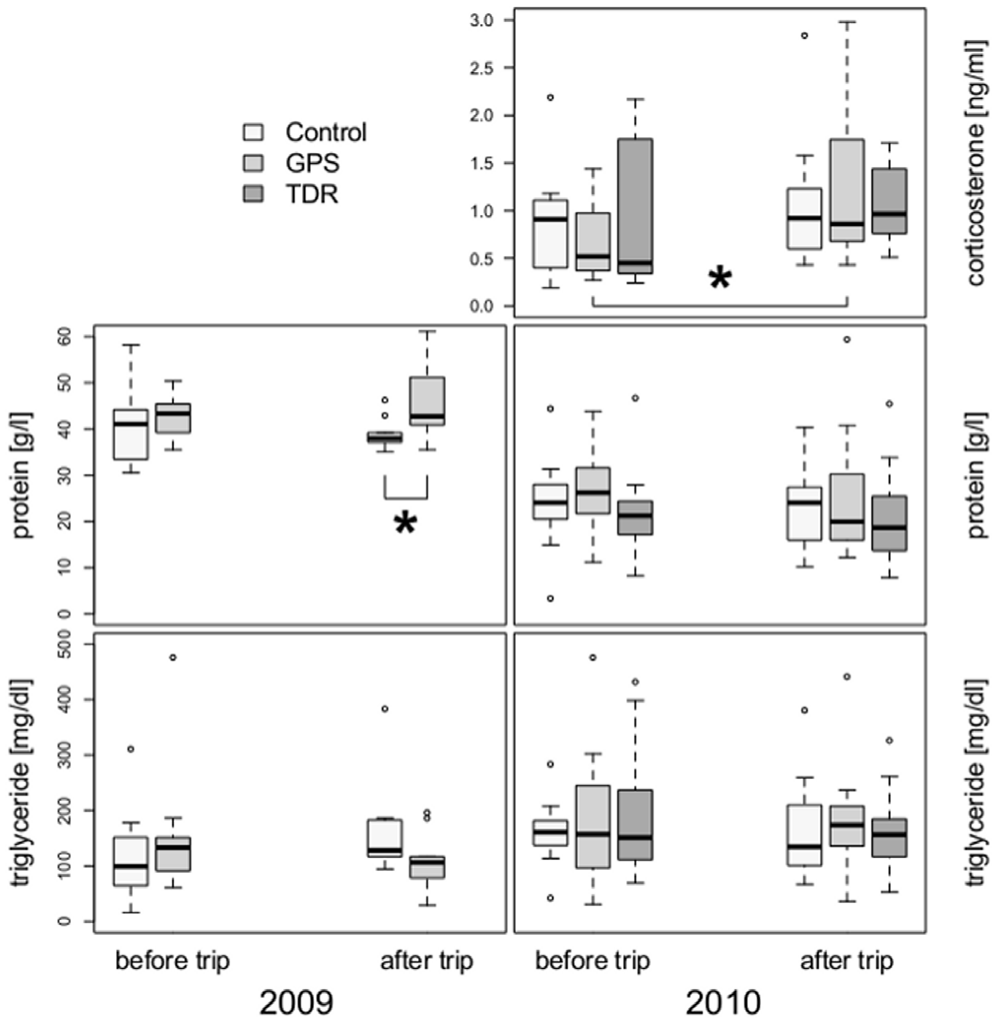
Figure 4. Physiological parameters in southern rockhopper penguins. Corticosterone, protein and triglyceride levels before and after foraging trips in females from New Island, equipped with GPS data loggers, miniature TDR dive loggers and from handled (but unequipped) control birds. The left panels represent 2009, the right panels represent 2010. The stars indicate significant differences between samples taken before and after foraging or between bird categories.

Baseline corticosterone levels measured in GPS, TDR and handled control birds in 2010 were consistently low and were not significantly different between bird categories before (chi-square = 4.7, df = 7, p = 0.693) or after foraging (chi-square = 8, df = 8, p = 0.434; Fig. 4). Nevertheless, GPS birds showed the highest corticosterone levels of all three bird categories after foraging (GPS: 1.218±0.224; TDR: 1.051±0.128; Control: 1.106±0.250 ng/ml; Fig. 4) and corticosterone levels increased significantly in GPS birds from before to after foraging (before: 0.684±0.112 ng/ml; V = 12, p = 0.034). No significant differences were found for handled controls (before: 0.870±0.208 ng/ml; V = 8, p = 0.098) or for TDR birds (before: 0.911±0.245; V = 17, p = 0.322) between corticosterone levels measured before and after foraging.

Protein levels in both breeding seasons did not change between measurements taken before and after the foraging trips within bird categories (GPS: 2009: V = 20, p = 0.492; 2010: V = 64, p = 0.502; Control: 2009: V = 35, p = 0.492; 2010: V = 46, p = 0.622; TDR: 2010: V = 60, p = 1.000; Fig. 4). In 2009, GPS birds had elevated protein levels after their foraging trips (45.88±2.54;) compared to handled control birds (Control: 38.98±1.03 g/l; W = 18.5, p = 0.019) but this was not the case in 2010 when compared to TDR and handled control birds (chi-square = 11, df = 11, p = 0.443; Fig. 4). Protein levels before their foraging trips were not significantly different between bird categories (2009: W = 40.5, p = 0.495; 2010: chi-square = 12, df = 12, p = 0.495). Protein levels in 2010 were overall higher than in 2009 (Fig. 4).

Triglyceride levels in both breeding seasons before and after foraging were not significant different between bird categories (2009: before: W = 30, p = 0.398; after: W = 61, p = 0.068; 2010: before: chi-square = 11, df = 11, p = 0.443; after: chi-square = 11, df = 11, p = 0.443) nor did we observe a change within bird categories between the two measurements (2009: handled controls: V = 26, p = 0.313; GPS: V = 16, p = 0.275; 2010: handled controls: V = 43, p = 0.791; GPS: V = 47, p = 0.761; TDR: V = 44, p = 0.389; Fig. 4). GPS birds had slightly lower triglyceride levels compared to handled control birds after foraging in 2009 (GPS: 105.57±17.18; Control: 166.73±32.89 mg/dl) but this was not found to be significant (see above).

**Figure 5:**
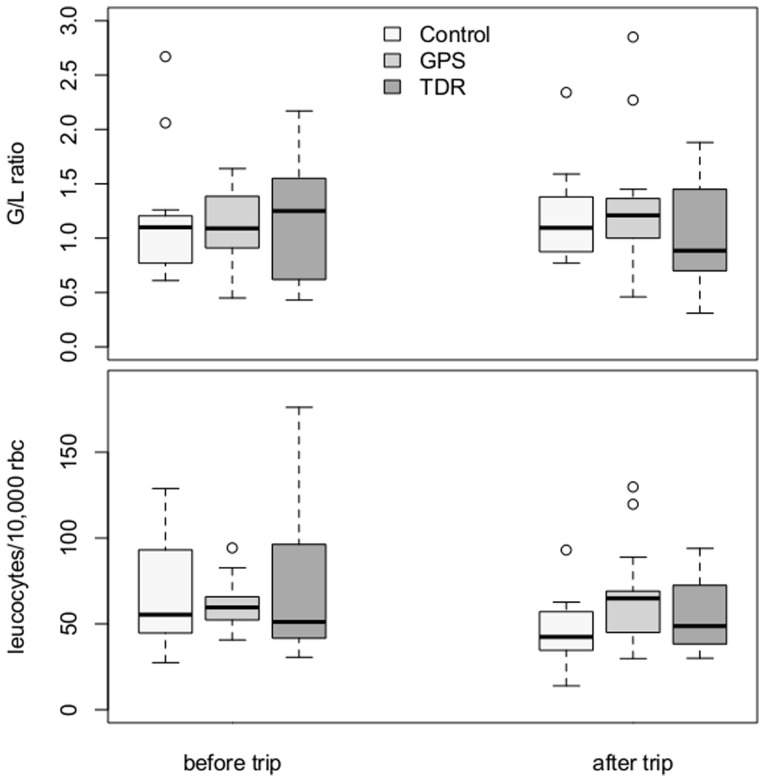
Blood parameters in southern rockhopper penguins. G/L ratio and leucocytes (per 10,000 RBC) before and after foraging trips by females from New Island, equipped with GPS data loggers, miniature TDR dive loggers and from handled control birds in 2010.

G/L ratio and leucocytes per 10,000 RBC did not change between measurements taken before and after foraging within bird categories (G/L: GPS: V = 43, p = 0.572; TDR: V = 61, p = 0.294; handled controls: V = 27, p = 0.380; leucocytes: GPS: V = 47, p = 0.489, TDR: V = 65, p = 0.463; handled controls: V = 63, p = 0.064). Differences between bird categories were also not found to be significant (G/L: before: chi-square = 11, df = 11, p = 0.443; after: chi-square = 10.2, df = 10, p = 0.422; leucocytes: before: chi-square = 11, df = 11, p = 0.443, after: chi-square = 11, df = 11, p = 0.443; Fig. 5).

## Discussion

In our study, most parameters showed no or little differences between the different bird categories. However, some parameters were significantly different for GPS birds, thus indicating negative impacts of GPS logger attachments. Foraging trip durations were similar between PIT control birds, handled control birds and logger-equipped birds in most breeding seasons. However, foraging trip duration was significantly longer for GPS birds in 2010 compared to other birds in the same breeding season and to GPS birds in previous breeding seasons. Rockhopper penguins during guard in 2010 also foraged further away from the colony, travelled larger distances and spent less time underwater compared to birds in the previous two breeding seasons. Logger and flipper band effects can become more obvious in years of unfavourable environmental conditions [9], [28], as birds are less capable to find food and thus to compensate for higher energy expenditure that they might experience while carrying the device [57]. However, Pietz et al. [58] states that transmitter effects may only be detectable during intermediate levels of environmental stress. During favourable years, even handicapped birds will be able to find enough food whereas during unfavourable years, all birds will show signs of stress [58]. Unfortunately, we have no direct measure of environmental conditions during the three breeding seasons of this study and it is therefore difficult to determine whether birds in 2010 were already compromised by unfavourable environmental conditions and thus more susceptible to logger effects than during the other breeding seasons. Breeding parameters are commonly used as indicators for food availability (see [59], [60] for overview) but breeding success, chick mass, average mass at arrival and average mass of females during the guard stage showed no differences between breeding seasons in our study (authors unpubl. data). However, a higher mortality between the seasons 2009/10 and 2010/11 was observed (authors unpubl. data), thus conditions might have been unfavourable before the onset of breeding.

As logger size might play an important role in the effects caused by the devices [16], [57], [61], we tested the effect of logger size using two different types of devices in 2010. Logger size did influence the diving behaviour of birds in our study as birds deployed with larger GPS data loggers presented more shallow dives compared to birds carrying miniature TDRs. Changes in dive behaviour are the most commonly observed effects of logger attachments in several penguin species (see Table 4 for overview). Little (*Eudyptula minor*) and Adélie penguins (*Pygoscelis adeliae*) dived shallower when carrying larger devices compared to birds with smaller loggers attached [16], [61]. Changes in dive depth frequencies, as observed in southern rockhopper penguins in our study, were also observed by Ropert-Coudert et al. [11] in king penguins (*Aptenodytes patagonicus*) carrying externally attached devices. According to Wilson [62], the maximum dive depths of Adélie and gentoo penguins (*Pygoscelis papua*) were correlated to the cross-sectional area of the devices, following the equation: max depth (m) = 166.5 - (0.08 × cross-sectional area in mm^2^). Applying this equation to our data, GPS birds should have reached only half the maximum depth of TDR birds. However, birds carrying smaller TDRs had only slightly greater maximum and mean dive depths and the deepest dives recorded were by GPS birds.

**Table 4:** Overview of logger impacts on different penguin species.

Categorie	Parameter		With impact		Without impact	
		Effect	Species	Reference	Species	Reference
Foraging behaviour	Trip duration	longer foraging trip duration	Southern Rockhopper P.	this study, [81]	Adélie P., Chinstrap P., King P.	[11], [18], [28], [61], [87]
			Adélie P.,Chinstrap P,Little P,Humboldt P.,Royal P.	[13], [16], [19], [76], [82]–[86]		
Diving behaviour	Divedepthfrequency	more shallow dives	Southern Rockhopper P.	this study		
	distribution		King P.	[11]		
	Dive depth	shallower dives	Adélie P., Little P.	[16], [61]	Southern Rockhopper P.	this study
		deeper dives	King P.	[11]	Southern Rockhopper P.	this study
	Number of dives	more dives	Little P.	[16]	Southern Rockhopper P.	this study
					Adélie P., King P.	[11], [61]
	Dive duration	shorter dive duration	Southern Rockhopper P.	this study	Adélie P.	[61]
			King P., Little P.	[11], [16]		
	Bottom time	shorter bottom time	Southern Rockhopper P.	this study	Little P.	[16]
	Post-dive duration	shorter post-dive duration	Southern Rockhopper P.	this study		
			King P.*	[11]		
	Dive efficiency	lesser dive efficiency	Southern Rockhopper P.	this study	Adélie P.	[61]
Physiological condition	Corticosteronelevels	increase	Southern Rockhopper P.	this study	Adélie P., King P.	[18], [19]
	Protein levels	increase	Southern Rockhopper P.	this study		
	Tryglyceride levels				Southern Rockhopper P.	this study
					Adélie P.	[19]

Effects are considered for externally attached devices (including flipper bands and harnesses) compared to smaller devices, internal devices or control birds. * King penguins showed longer post-dive durations between deep dives, this was not found for rockhopper penguins in this study (see results).

Besides alterations of foraging and diving behaviour, handling and logger attachments can have effects on physiological parameters [15], [19]–[21], [63]. Even birds that seem to be calm can be stressed while being handled or disturbed [4], [64], [65] and the pure presence of a human close to a bird can cause alterations in stress hormones [66]. In our study, little impact was measured in physiological parameters due to logger attachments. GPS birds did show elevated protein levels in 2009 though when compared to handled control birds after foraging. We also found a significant increase in corticosterone levels in 2010 from before to after foraging in GPS birds, although GPS birds had the lowest median corticosterone level in 2010 after foraging. Significant results, however, could be influenced by multiple testing as we conducted several tests on the same data set [67]. The large variation in baseline corticosterone values observed in our study might be due to individual differences in behaviour, as, for example, more aggressive birds show higher baseline corticosterone values [68], [69]. In king penguins, corticosterone levels were not found to be related to food acquisition [18] but several studies have found baseline corticosterone levels to be related to food-related stress in other seabirds ([70]: Wilson’s storm-petrels (*Oceanites oceanicus*), [30]: common murres (*Uria aalge*)). Changes in protein and triglyceride levels can also be attributed to food intake and the nutritional state of the animal [19], [23], [70]. Additionally, protein levels are known to increase under physical exercise [24]. However, Navarro et al. [15] found no significant differences in plasma protein and triglycerides of handicapped compared to non-handicapped Cory’s shearwaters (*Calonectris diomedea*) and no significant differences in plasma triglyceride and uric acid (an indicator for protein breakdown) were found in Adélie penguins even though logger-equipped birds did present longer foraging trips [19]. Similarly, longer foraging trips and less foraging success did not lead to changes in physiological parameters in our study.

While the number of leucocytes per 10,000 RBC so far has been mainly shown to decline during handling stress (e.g. [20]), the G/L ratio in southern rockhopper penguins is related to body condition, and higher values indicated stress during fasting periods [54]. In fact, other studies showed that the equivalently used H/L ratio could be more sensitive to certain environmental and chronic stressors than changes in baseline corticosterone [21], [71], [72]. Nevertheless, our results show no increase in G/L ratios and no decrease in leucocyte numbers in control or logger equipped birds. Thus, potentially stressful handling and logger attachment, as detected by elevated cortiscosterone and protein levels, seem not to have affected leucocyte counts. Similarly, Jakubas et al. [73] did not find differences in H/L ratios in little auks (*Alle alle*) breeding under different oceanographic conditions. Birds compensated for lower food abundance and changes in H/L ratio might only become detectable once food sources are below a certain threshold level [73].

Overall, effects of handling and logger attachment seem to be rather small when considering foraging behaviour and physiological parameters although GPS devices did influence the diving behaviour and foraging success in one of the breeding seasons, likely as a consequence of the GPS devices’ larger cross-sectional area, and might have caused birds to forage longer during one of the breeding seasons. Many studies have found no effect of logger attachments and flipper bands during single seasons or during short-term deployments ([28], [74], [75], but see [7], [10]) but long-term studies have shown increased corticosterone levels after year-long deployments with geolocators [63] and reduced survival in birds equipped with flipper bands [9]. In our study, birds were only deployed for one foraging trip in 2009 and 2010, usually not lasting longer than one day, and for three days in 2008 (up to three foraging trips; [35]), thus impact of logger attachment was to be expected lower than in studies where birds carried devices for several weeks [13], [76]. Short deployments might not be felt as chronic stress by the birds, thus explaining the little effects on physiological parameters [19]. Short term deployments of PTTs on wandering albatrosses (*Diomedea exulans*) have not been found to negatively influence demographic parameters ([77], but see [7]) and resighting rates of previously logger-equipped rockhopper penguins in our study were similar to the overall resighting rate of PIT tagged birds (authors unpubl. data).

## Conclusions

Studies on flying birds indicate that devices should not exceed 3% of the body mass ([74], but see [7], [78]) for further considerations) but for swimming and diving birds, the cross-sectional dimensions seem to be more important than mass [12], [16], [61]. Even though GPS data loggers weighed less than 3% of the rockhopper body masses, they had relatively large cross-sectional areas compared to miniature TDRs. Significant differences in the dive depth frequencies between GPS and TDR birds can be most probably related to the increased drag due to the larger size of our GPS devices [79]. Besides potentially causing higher energy expenditure for the birds [80], it also reveals that dive parameters measured by externally (and possibly also internally; see [14]) attached devices might not represent the birds’ normal diving behaviour, thus not representing “real” dive data, although this could be derived through extrapolation (see [61]). Longer foraging trip durations, foraging further away from the colony and increased baseline corticosterone levels were only observed in 2010. These factors might be linked as birds foraging further away from the island would spend more time at sea and possibly have higher foraging costs, which could explain the high corticosterone levels. Differences observed in 2010 could be related to unfavourable environmental conditions, which might have also further enhanced the differences found in the dive parameters between GPS and TDR birds.

One-day (or very short) logger attachments have little effects on the overall breeding activity of the birds even though they may cause lower energetic gain during the trip [16]. We are therefore confident that the use of externally attached devices on southern rockhopper penguins in our study has not jeopardised the survival of the birds but device sizes should be reduced in future studies to eliminate the effects we did find on certain foraging, dive and physiological parameters.

## References

[1] Cooke SJ, Hinch SG, Wikelski M, Andrews RD, Kuchel LJ, et al (2004) Biotelemetry: a mechanistic approach to ecology. Trends Ecol Evol 19: 334–343. 16701280 10.1016/j.tree.2004.04.003

[2] Ropert-Coudert Y, Wilson RP (2005) Trends and perspectives in animal-attached remote sensing. Front Ecol and Environ 3: 437–444.

[3] Wilson RP, Vandenabeele S (2012) Technological innovation in archival tags used in seabird research. Mar Ecol Prog Ser 451: 245–262.

[4] Hawkins P (2004) Bio-logging and animal welfare: practical refinements. Mem Natl Inst Polar Res 58: 58–68.

[5] Wilson RP, McMahon CR (2006) Measuring devices on wild animals: what constitutes acceptable practice? Front Ecol and Environ 4: 147–154.

[6] Casper RM (2009) Guidelines for the instrumentation of wild birds and mammals. Anim Behav 78: 1477–1483.

[7] Barron DG, Brawn JD, Weatherhead PJ (2010) Meta-analysis of transmitter effects on avian behaviour and ecology. Methods Ecol Evol 1: 180–187.

[8] Murray DL (2000) A critical review of the effects of marking on the biology of vertebrates. In: Boitani L, Fuller T, editors. Research Techniques in Animal Ecology, Controversies and Consequences. New York: Columbia University Press. 15–64.

[9] Saraux C, Le Bohec C, Durant JM, Viblanc VA, Gauthier-Clerc M, et al (2011) Reliability of flipper-banded penguins as indicators of climate change. Nature 469: 203–206. 21228875 10.1038/nature09630

[10] Vandenabeele SP, Wilson RP, Grogan A (2011) Tags on seabirds: how seriously are instrument-induced behaviours considered? Anim Welf 20: 559–571.

[11] Ropert-Coudert Y, Bost C-A, Handrich Y, Bevan RM, Butler PJ, et al (2000) Impact of externally attached loggers on the diving behaviour of the king penguin. Physiol Biochem Zool 73: 438–445. 11009397 10.1086/317743

[12] Wilson RP, Grant WS, Duffy DC (1986) Recording devices on free-ranging marine animals: does measurement affect foraging performance? Ecology 67: 1091–1093.

[13] Hull CL (1997) The effect of carrying devices on breeding royal penguins. Condor 99: 530–534.

[14] Beaulieu M, Ropert-Coudert Y, Le Maho Y, Ancel A (2010) Is abdominal implantation of devices a good alternative to external attachment? A comparative study in Adélie penguins. J Ornithol 151: 579–586.

[15] Navarro J, González-Solís J, Viscor G, Chastel O (2008) Ecophysiological response to an experimental increase in wing loading in a pelagic seabird. J Exp Mar Bio Ecol 358: 14–19.

[16] Ropert-Coudert Y, Knott N, Chiaradia A, Kato A (2007) How do different data logger sizes and attachment positions affect the diving behaviour of little penguins? Deep Sea Res Part 2 Top Stud Oceanogr 54: 415–423.

[17] Ponganis PJ (2007) Bio-logging of physiological parameters in higher marine vertebrates. Deep Sea Res Part 2 Top Stud Oceanogr 54: 183–192.

[18] Angelier F, Giraudeau M, Bost C-A, Le Bouard F, Chastel O (2009) Are stress hormone levels a good proxy of foraging success? An experiment with king penguins, *Aptenodytes patagonicus* . J Exp Biol 212: 2824–2829. 19684217 10.1242/jeb.027722

[19] Beaulieu M, Spée M, Lazin D, Ropert-Coudert Y, Le Maho Y, et al (2010) Ecophysiological response of Adélie penguins facing an experimental increase in breeding constraints. J Exp Biol 213: 33–39. 20008359 10.1242/jeb.035378

[20] Davis AK (2005) Effect of handling time and repeated sampling on avian white blood cell counts. J Field Ornithol 76: 334–338.

[21] Quillfeldt P, McGill RAR, Furness RW, Möstl E, Ludynia K, et al (2012) Impact of miniature geolocation loggers on a small petrel, the thin-billed prion *Pachyptila belcheri* . Mar Biol 159: 1809–1816.

[22] Jenni-Eiermann S, Jenni L (1996) Metabolic differences between the postbreeding, moulting and migratory periods in feeding and fasting passerine birds. Funct Ecol 10: 62–72.

[23] Jenni-Eiermann S, Jenni L (1998) What can plasma metabolites tell us about the metabolism, physiological state and condition of individual birds? An overview. Biol Cons Fauna 102: 312–319.

[24] Ots I, Murumägi A, Hõrak P (1998) Haematological health state indices of reproducing great tits: methodology and sources of natural variation. Funct Ecol 12: 700–707.

[25] Nelson RJ (2005) An introduction to behavioural endocrinology. 3^rd^ Edition. Sunderland: Sinauer Associates, Inc. Publishers.

[26] Selye H (1950) Stress. Montreal: Acta.

[27] Wingfield JC, Smith JP, Farner DS (1982) Endocrine response of white-crowned sparrows to environmental stress. Condor 84: 399–409.

[28] Ballard G, Ainley DG, Ribic CA, Barton KR (2001) Effect of instrument attachment and other factors on foraging trip duration and nesting success of Adélie penguins. Condor 103: 481–490.

[29] Buck CL, O’Reilly KM, Kildaw SD (2007) Interannual variability of black-legged kittiwake productivity is reflected in baseline plasma corticosterone. Gen Comp Endocrinol 150: 430–436. 17161400 10.1016/j.ygcen.2006.10.011

[30] Kitayska AS, Piatt JF, Wingfield JC (2007) Stress hormones link food availability and population processes in seabirds. Mar Ecol Prog Ser 352: 245–258.

[31] Gladbach A, Gladbach DJ, Quillfeldt P (2010) Variations in leucocyte profiles and plasma biochemistry are related to different aspects of parental investment in male and female upland geese *Chloephaga picta leucoptera* . Comp Biochem Physiol A 159: 269–277. 10.1016/j.cbpa.2010.02.012 20176125

[32] Strange IJ, Catry P, Strange G, Quillfeldt P (2007) New Island, Falkland Islands. A South Atlantic wildlife sanctuary for conservation management. New Island Conservation Trust. Stanley, Falkland Islands.

[33] Strange IJ (1982) Breeding ecology of the rockhopper penguin (*Eudyptes crestatus*) in the Falkland Islands. Le Gerfaut 72: 137–188.

[34] Williams TD (1995) The Penguins - Spheniscidae. Oxford University Press, Oxford, UK.

[35] Masello JF, Mundry R, Poisbleau M, Demongin L, Voigt CC, et al (2010) Diving seabirds share foraging space and time within and among species. Ecosphere 1: art19.

[36] Ryan PG, Petersen SL, Peters G, Grémillet D (2004) Field testing GPS loggers on marine predators; effects of precision, resolution and sampling rate on foraging tracks of African penguins. Mar Biol 145: 215–223.

[37] Wilson RP, Pütz K, Peters G, Culik BM, Scolaro JA, et al (1997) Long-term attachment of transmitting and recording devices to penguins and other seabirds. Wildl Soc Bull 25: 101–106.

[38] Poisbleau M, Demongin L, van Noordwijk HJ, Strange IJ, Quillfeldt P (2010) Sexual dimorphism and use of morphological measurements to sex adults, immatures and chicks of rockhopper penguin. Ardea 98: 217–224.

[39] Ellenberg U, Mattern T, Houston DM, Davis LD, Seddon PJ (2012) Previous experiences with humans affect responses of Snares penguins to experimental disturbance. J Ornithol DOI 10.1007/s10336-011-0780-4. In press.

[40] Clarke J, Kerry K (1998) Implanted transponders in penguins: implantation, reliability, and long-term effects. J Field Ornithol 69: 149–159.

[41] Renner M, Davis LS (2000) Marking penguins with implanted transponders. Notornis 47: 163–165.

[42] Gauthier-Clerc M, Gendner J-P, Ribic CA, Fraser WR, Woehler EJ, et al (2004) Long-term effects of flipper bands on penguins. Proc R Soc Lond B 271: S423–S426. 10.1098/rsbl.2004.0201 PMC1810082 15801593

[43] Schroeder J, Cleasby IR, Nakagawa S, Ockendon N, Burke T (2011) No evidence for adverse effects on fitness of fitting passive integrated transponders (PITs) in wild house sparrows *Passer domesticus* . J Avian Biol 42: 271–275.

[44] R Development Core Team (2009) R: a language and environment for statistical computing. R Foundation for Statistical Computing, Vienna.

[45] Pinheiro J, Bates D, DebRoy S, Sarkar D, R Development Core Team (2011) nlme: Linear and nonlinear mixed effect models. R package version 3.1–101.

[46] Cherel Y, Tremblay Y, Guinard E, Georges JY (1999) Diving behaviour of female northern rockhopper penguins, *Eudyptes chrysocome moseleyi*, during the brooding period at Amsterdam Island (Southern Indian Ocean). Mar Biol 134: 375–385.

[47] Mattern T, Ellenberg U, Houston DM, Davis LS (2007) Consistent foraging routes and benthic foraging behaviour in yellow-eyed penguin. Mar Ecol Prog Ser 343: 295–306.

[48] Wilson RP, Pütz K, Charrassin J-B, Lage J (1995) Artifacts arising from sampling intervals in dive depth studies or marine endotherms. Polar Biol 15: 575–581.

[49] Tremblay Y, Cherel Y (2000) Benthic and pelagic dives: a new foraging behaviour in rockhopper penguins. Mar Ecol Prog Ser 204: 257–267.

[50] Ydenberg RC, Clark CW (1989) Aerobiosis and anaerobiosis during diving by western grebes: an optimal foraging approach. J Theor Biol 139: 437–449.

[51] Ruiz G, Rosenmann M, Novoa FF, Sabat P (2002) Hematological parameters and stress index in rufous-collared sparrows dwelling in urban environments. Condor 104: 162–166.

[52] Masello JF, Quillfeldt P (2004) Are haematological parameters related to body condition, ornamentation and breeding success in wild burrowing parrots *Cyanoliseus patagonus* ? J Avian Biol 35: 445–454.

[53] Merino S, Martínez J, Møller AP, Sanabria L, de Lope F, et al (1999) Phytohaemagglutinin injection assay and physiological stress in nestling house martins. Anim Behav 58: 219–222. 10413560 10.1006/anbe.1999.1127

[54] Dehnhard N, Poisbleau M, Demongin L, Quillfeldt P (2011) Do leucocyte profiles reflect temporal and sexual variation in body condition over the breeding cycle in southern rockhopper penguins? J Ornithol 152: 759–768.

[55] Hawkey CM, Dennet PB (1989) A colour atlas of comparative veterinary haematology. Ipswich :Wolfe.

[56] Hoi-Leitner M, Romero-Pujante M, Hoi H, Pavlova A (2001) Food availability and immune capacity in serin (*Serinus serinus*) nestlings. Behav Ecol Sociobiol 49: 333–339.

[57] Culik B, Wilson RP (1992) Field metabolic rates of instrumented Adélie penguins using double-labelled water. J Comp Physiol B 162: 567–573.

[58] Pietz PJ, Krapu GL, Greenwood RJ, Lokemoen JT (1993) Effects of harness transmitters on behaviour and reproduction of wild mallards. J Wildl Manage 57: 696–703.

[59] Piatt JF, Harding AMA, Shultz M, Speckman SG, van Pelt TI, et al (2007) Seabirds as indicators of marine food supplies: Cairns revisited. Mar Ecol Prog Ser 352: 221–234.

[60] Grémillet D, Charmantier A (2010) Shifts in phenotypic plasticity constrain the value of seabirds as ecological indicators of marine ecosystems. Ecol Appl 20: 1498–1503. 20945754 10.1890/09-1586.1

[61] Ropert-Coudert Y, Wilson RP, Yoda K, Kato A (2007) Assessing performance constraints in penguins with externally-attached devices. Mar Ecol Prog Ser 333: 281–289.

[62] Wilson RP (1989) Diving depths of gentoo *Pygoscelis papua* and Adélie *P. adeliae* penguins at Esperanza Bay, Antarctic Peninsula. Cormorant 17: 1–8.

[63] Elliott KH, McFarlane-Tranquilla L, Burke CM, Hedd A, Montevecchi WA, et al (2012) Year-long deployments of small geolocators increase corticosterone levels in murres. Mar Ecol Prog Ser 466: 1–7.

[64] Culik B, Adelung D, Woakes AJ (1990) The effect of disturbance on the heart rate and behaviour of Adélie pengions (*Pygoscelis adeliae*) during the breeding season. In: Kerry KR, Hempel G, editors. Antarctic Ecosystems: Ecological Change and Conservation. Berlin: Springer. 177–182.

[65] Le MahoY, Karmann H, Briot D, Handrich Y, Robin JP, et al (1992) Stress in birds due to routine handling and a technique to avoid it. Am J Physiol 263: 775–781. 10.1152/ajpregu.1992.263.4.R775 1415787

[66] Fowler GS (1990) Behavioral and hormonal responses of Magellanic penguins (*Spheniscus magellanicus*) to tourism and nest site visitation. Biol Conserv 90: 143–149.

[67] Young SS, Karr A (2011) Deming, data and observational studies. Significance 8: 116–120.

[68] Ellenberg U, Setiawan AN, Cree A, Houston DM, Deeon PJ (2007) Elevated hormonal stress response and reduced reproductive output in yellow-eyed penguins exposed to unregulated tourism. Gen Comp Endocrinol 152: 54–63. 17400221 10.1016/j.ygcen.2007.02.022

[69] Ellenberg U, Mattern T, Seddon PJ (2009) Habituation potential of yellow-eyed penguins depend on sex, character and previous experience with humans. Anim Behav 77: 289–296.

[70] Quillfeldt P, Masello JF, Möstl E (2004) Blood chemistry in relation to nutrition and ectoparasite load in Wilson’s storm-petrels *Oceanites oceanicus* . Polar Biol 27: 168–176.

[71] Vleck CM, Vertalino N, Vleck D, Bucher TL (2000) Stress, corticosterone, and heterophil to lymphocyte ratios in free-living Adélie penguins. Condor 102: 392–400.

[72] Müller C, Jenni-Eiermann S, Jenni L (2011) Heterophils/Lymphocytes-ratio and circulating corticosterone do not indicate the same stress imposed on Eurasian kestrel nestlings. Funct Ecol 25: 566–576.

[73] Jakubas D, Gluchowska M, Wojczulanis-Jakubas K, Karnovsky NJ, Keslinka L, et al (2011) Foraging effort does not influence body condition and stress levels in little auks. Mar Ecol Prog Ser 432: 277–290.

[74] Phillips RA, Xavier JC, Croxall JP (2003) Effects of satellite transmitters on albatrosses and petrels. Auk 120: 1082–1090.

[75] Hampton SL, Ryan PG, Underhill LG (2009) The effect of flipper banding on the breeding success of African penguins *Speniscus demersus* at Boulders Beach, South Africa. Ostrich 80: 77–80.

[76] Wilson RP, Coria NR, Spairani HJ, Adelung D, Culik B (1989) Human-induced behaviour in Adélie penguins *Pygoscelis adeliae* . Polar Biol 10: 77–80.

[77] Barbraud C, Weimerskirch H (2012) Assessing the effect of satellite transmitters on the demography of the wandering albatross *Diomedea exulans* . J Ornithol 153: 375–383.

[78] Vandenabeele SP, Shepard EL, Grogan A, Wilson RP (2012) When three per cent may not be three per cent; device-equipped seabirds experience variable flight constraints. Mar Biol 159: 1–14.

[79] Bannasch R, Wilson RP, Culik B (1994) Hydrodynamic aspects of design and attachment of a back-mounted device in penguins. J Exp Biol 194: 83–96. 9317385 10.1242/jeb.194.1.83

[80] Culik B, Wilson RP (1991) Swimming energetics and performance of instrumented Adélie penguins (*Pygoscelis adeliae*). J Exp Biol 158: 355–368.

[81] Pütz K, Smith JG, Ingham RJ, Lüthi BH (2003) Satellite tracking of male rockhopper penguins *Eudyptes chrysocome* during the incubation period at the Falkland Islands. J Avian Biol 34: 139–144.

[82] Croll DA, Osmek SD, Bengston JL (1991) An effect of instrument attachment on foraging trip duration in chinstrap penguins. Condor 93: 777–779.

[83] Watanuki Y, Mori Y, Naito Y (1992) Adélie penguin parental activities and reproduction: effects of device size and timing of its attachment during chick rearing period. Polar Biol 12: 539–544.

[84] Miller GD, Davis LS (1993) Foraging flexibility of Adélie penguins *Pygoscelis adeliae*: Consequences for an indicator species. Biol Conserv 63: 223–230.

[85] Taylor SS, Leonard ML, Boness DJ (2001) Foraging trip duration increases for Humboldt penguins tagged with recording devices. J Avian Biol 32: 369–372.

[86] Dugger KM, Ballard G, Ainley DG, Barton KJ (2006) Effects of flipper bands on foraging behavior and survival of Adélie penguins (*Pygoscelis adeliae*). Auk 123: 858–869.

[87] Croll DA, Jansen JK, Goebel ME, Boveng PL, Bengtson JL (1996) Foraging behavior and reproductive success in chinstrap penguins: the effects of transmitter attachment. J Field Ornithol 67: 1–9.

